# Coexistence of endometriosis and human papilloma virus: A systematic review and meta-analysis

**DOI:** 10.1016/j.nmni.2026.101802

**Published:** 2026-06-19

**Authors:** Marzieh Saei Ghare Naz, Mahbanoo Farhadi-Azar, Zahra Marzban Rad, Shahla Noori Ardebili, Niloofar Harati, Niloofar Ghassemi Firouzabadi, Fatemeh Jayervand, Fahimeh Ramezani Tehrani

**Affiliations:** a Reproductive Endocrinology Research Center, Research Institute for Endocrine Molecular Biology, Research Institute for Endocrine Sciences, Shahid Beheshti University of Medical Sciences, Tehran, Iran; b Department of Gynecologic Oncological, School of Medicine, Shahid Beheshti University of Medical Sciences, Tehran, Iran; c Department of Gynecology and Obstetrics, School of Medicine, Iran University of Medical Sciences, Tehran, Iran; d Foundation for Research & Education Excellence, Vestavia Hills, AL, USA

**Keywords:** Endometriosis, Human papilloma virus, Review, Women

## Abstract

**Purpose:**

Recently, several studies have explored the potential association between endometriosis and human papillomavirus. This study aimed to investigate whether women with endometriosis are prone to human papillomavirus and whether women with human papillomavirus are prone to endometriosis.

**Method:**

In this systematic review and meta-analysis, the search was performed on Web of Science, Scopus, and PubMed from inception to August 2025. The quality assessment was performed using the Newcastle–Ottawa Scale. The pooled effect size (OR or proportion) was calculated using random-effects models. Egger's test and Begg's test were used for assessing publication bias.

**Result:**

In total 12 studies with 38265 participants were included. The pooled prevalence estimates of human papillomavirus regardless of the type among women with endometriosis was 29.73% [95% confidence interval (CI): 14.218 to 48.149; I^2^:93.93%, n = 8). Using a random-effects model, the estimated odds ratio of endometriosis in women with positive human papillomavirus was 1.226 (95% CI: 0.87–1.724; p = 0.243; I^2^:71.06%, n = 4). The estimated pooled odds ratio of human papillomavirus regardless of the type among women with endometriosis was 1.218 (95% CI: 0.569 to 2.608; I^2^ = 73.73%; p = 0.507, n = 7).

**Conclusion:**

Although this study indicates a higher prevalence of human papillomavirus in women with endometriosis, the pooled odds ratios showed that human papillomavirus and endometriosis do not significantly influence the risk of developing each other. The pooled prevalence estimate carries high heterogeneity, which limits interpretation. Additional research is necessary to investigate the relationship between human papillomavirus and endometriosis.

**Prospero registration code:**

CRD420251132982.

## 1 Introduction

Endometriosis (EM) is a chronic health condition, and its prevalence, based on clinical data studies and symptomatic patient data, was reported to be 6.6% and 21%, respectively [1]. It is characterized by the presence of endometrial-like tissue outside the uterus [2]. EM is associated with pelvic pain, infertility, and potentially involvement of multiple organs [3]. Most international guidelines recommend a non-surgical approach for the diagnosis of EM, while surgical visualization and histological verification confirm the diagnosis [3]. Multiple factors, including autoimmune, congenital, environmental, and epigenetic factors, can contribute to the development of EM [4]. Additionally, evidence shows that inflammatory factors play a crucial role in the pathophysiology of EM [5].

A recent study on the association between EM and the microbiome revealed that EM was associated with an increased presence of Proteobacteria, Enterobacteriaceae, Streptococcus spp., and *Escherichia coli* [6]. Evidence also supports the potential causal relationship between EM and pelvic inflammatory disease (PID) [7]. By contrast, a cohort study showed that women with PID were at a threefold higher risk of developing EM [8]. The human papillomavirus (HPV) in women is considered the most common sexually transmitted infection (STI) [9]. The worldwide pooled any-HPV prevalence among women over 50 years reported about 11·70% [10]. According recent studies among reproductive age women HPV is also highly prevalent [11,12].

Recently, some studies have focused on the hypothetical association between EM and HPV; even a few studies have reported elevated rates of HPV DNA in endometriotic cells compared to control tissues, suggesting a potential cofactor role in the development or exacerbation of the disease [13]. One study reported a significantly higher rate of high-risk HPV in ovarian endometriotic lesions compared to healthy ovarian tissues, supporting the hypothesis of a viral contribution to endometriotic lesion formation [14]. However, not all studies have confirmed this association; for instance, a recent study by Hong et al. demonstrated a low prevalence of HPV in endometriosis cases, but methodological limitations, such as a small sample size and heterogeneity of tissue types, may have affected their conclusions [15]. Previous studies' findings raise important questions about whether HPV acts as a direct or indirect cofactor in EM via inflammatory and immune-mediated pathways.

Endometriosis is a chronic inflammatory and immune-mediated condition [16,17]. There is also evidence that supports the theory of co-regulation of HPV infection by the immune system [18]. If strong causal evidence emerged that HPV contributes to EM pathogenesis or vice versa, this would have multiple implications in screening and prevention of HPV and EM. Despite growing interest in a possible association between HPV and EM, current evidence does not conclusively demonstrate that HPV is a primary driver of EM or that EM is a primary driver of HPV. Thus, this hypothetical association remains inconclusive and still needs more studies. To achieve a clearer understanding of the potential association, the present study is designed as a meta-analysis.

## 2 Methods

This systematic review and meta-analysis was conducted in accordance with the Preferred Reporting Items for Systematic Reviews and Meta-Analyses (PRISMA 2020) statement [19]. This study was registered in PROSPERO (registration code: CRD420251132982).

### 2.1 Eligibility criteria

The eligibility criteria were defined based on the PICO items according to the study questions (Table 1).

**Table 1:** PICO items of study.

Study Question	Population	Exposure	Comparator	Outcomes	Study designs
Are women with HPV at risk of EM? Or How much is prevalence of EM among women with HPV?	Women of reproductive age diagnosed with HPV	Laboratory-confirmed HPV infection	Women without HPV	Prevalence, odds ratio (OR), relative risk (RR), or hazard ratio (HR) of EM in women with HPV compared with controls	Observational studies (cross-sectional, case-control, cohort) and randomized controlled trials (RCTs) reporting relevant data
Are women with EM at risk of HPV? Or How much is prevalence of HPV among women with EM?	Women of reproductive age diagnosed with EM	EM according to recognized definition (non-surgical and surgical)	Women with EM but without HPV	Prevalence, odds ratio (OR), relative risk (RR), or hazard ratio (HR) of HPV in women with EM compared with controls	Observational studies (cross-sectional, case-control, cohort) and randomized controlled trials (RCTs) reporting relevant data

### 2.2 Search strategy

The search was performed on the following electronic databases from inception to August 2025: Web of Science, Scopus, and PubMed. There were no restrictions on publication date. However, due to limitations on comprehensive multilingual screening and data extraction, the search of studies was limited to the English language.

The search strategy was combining controlled vocabulary (e.g., MeSH) and free-text terms related to EM and HPV. An example PubMed search string is:

#1:

(Disease, Sexually Transmitted [Title/Abstract]) OR (Diseases, Sexually Transmitted[Title/Abstract])) OR (Sexually Transmitted Disease[Title/Abstract])) OR (Sexually Transmitted Infections[Title/Abstract])) OR (Infection, Sexually Transmitted[Title/Abstract])) OR (Infections, Sexually Transmitted[Title/Abstract])) OR (STIs[Title/Abstract])) OR (STI[Title/Abstract])) OR (HPV[Title/Abstract])) OR (Human Papillomavirus Virus[Title/Abstract])) OR (Papillomavirus Virus, Human[Title/Abstract])) OR (Virus, Human Papillomavirus[Title/Abstract])) OR (Human Papilloma Virus[Title/Abstract])) OR (HPV Human Papillomavirus[Title/Abstract])) OR (Human Papillomavirus, HPV[Title/Abstract])) OR (HPV, Human Papillomavirus Viruses[Title/Abstract]))

#2:

((Endometriosis [Title/Abstract]) OR (Endometrioses [Title/Abstract])) OR (Endometrioma [Title/Abstract])) OR (Endometriomas [Title/Abstract]))

#3:

#1 AND #2.

Reference lists of included studies and relevant reviews were also screened manually for additional eligible studies.

### 2.3 Study selection

Two independent reviewers (M.S.G and M.F.A) screened titles and abstracts, followed by full-text review to determine eligibility. Discrepancies resolved by consensus or by a third one.

### 2.4 Data extraction

Using a standardized form, two reviewers independently extracted:

Study characteristics (authors, year, country, study design, setting, sample size, diagnostic criteria), participant characteristics (age, BMI), effect measures (prevalence, OR, RR, HR) with 95% confidence intervals, and adjustments for confounders.

A summary table outlining the methods used, such as (e.g., ultrasound criteria, histological confirmation) for EM diagnosis. Also, a summary of the HPV detection techniques, such as (e.g., PCR-based methods, genotyping specific methods, etc.).

### 2.5 Risk of bias assessment

Observational studies were assessed using the Newcastle–Ottawa Scale (NOS) by two reviewers. This tool evaluated the selection, comparability, and exposure/outcome items in the included studies. The maximum score is 9. The high-quality studies scored 7-9 stars, moderate-quality studies scored 4-6 stars, and those with 0-3 stars were considered low quality. Disagreements were resolved through discussion.

### 2.6 Statistical analysis

Prevalence estimates were calculated to describe the overall frequency of each outcome across the included studies. Measures of association (odds ratios) were synthesized to evaluate the relationship between the exposures and the outcome. The pooled effect size (OR or proportion) was calculated using random-effects models (DerSimonian–Laird method) to account for between-study heterogeneity. If studies reported prevalence only, we calculated effect sizes where possible. Heterogeneity was determined using the Cochran's Q statistic, and its magnitude was evaluated using the I2 statistic. An I^2^ value above 50% was considered substantial heterogeneity; for these cases, the random-effects model was applied.

Heterogeneity was quantified using Cochran's Q statistic and the I^2^ statistic. Funnel plots, Egger's test, and Begg's test were used to assess publication bias in the included studies of the meta-analysis.

To assess the robustness of the pooled estimates, a sensitivity analysis was conducted by sequentially excluding studies that appeared to be outliers. After removing the study (or studies) with markedly different effect sizes, the meta-analysis was re-run to examine the influence of their exclusion on the overall results.

Analyses were performed using MedCalc® Statistical Software version 22.014, Ltd, Ostend, Belgium; https://www.medcalc.org. A p-value ≤0.05 was considered a significant level.

## 3 Results

**Fig. 1:**
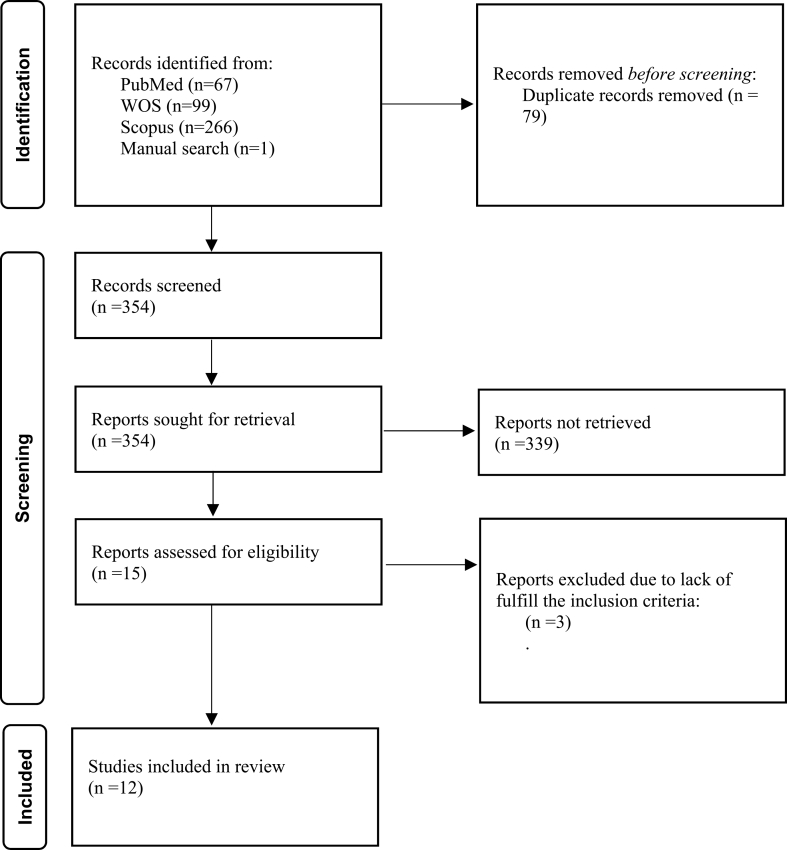
Study selection.

Following an advanced search, 433 records were included; after removing duplications (n = 79), 354 records were screened for title and abstract. After removing irrelevant records, 15 records underwent a comprehensive full-text evaluation. Finally, 12 articles satisfied the inclusion criteria and were incorporated into the meta-analysis. (Fig. 1).

Among included studies 7 of them (Heidarpour et al. [20], Hong et al. [21], Oppelt et al. [22], Azizvakili et al. [23], Vestergaard et al. [24], Okyay et al., [25], Rocha et al. [26]) were evaluated the HPV in women with and without EM (EM = 454 women, non-EM = 2165 women). One of the studies (Moslehi et al., [27]) assessed HPV in women with EM (n = 81) without non-EM group. The rest of studies (Zullo et al. [28], Hsu et al. [29], Hsu et al. [30], Wei et al. [31]) assessed the EM in women with positive HPV (n = 12582 women) compared to women with negative HPV (n = 22983 women).

All studies were observational studies that were published between 2010 and 2023. 7 studies in Asia, 3 studies in Europe and 1 in America were performed.

Table 2 shows the characteristics of included studies.

**Table 2:** Characteristics of included studies.

Author, year, Ref	Country	Sample size		Type		Participants characteristics	Diagnostic criteria		Findings		Quality assessments
		EM	HPV	EM	HPV		EM	HPV	EM	HPV	
Heidarpour M et al., 2017 [14]	Iran	50	NA	ovarian	16, 18, 31, 33, 35, 39, 45, 52, 56, 58, and 59	Patients with endometriosis (High-risk HPV+):42.82 ± 7.41 High-risk HPV–: 43.88 ± 7.49 BMI: NA	Medical files and Samples of ovarian endometriosis had been obtained by laparoscopic surgery	HPV DNA detection was performed using general HPV primers.	NA	High-risk HPV infection was detected in 13 (26%) of the samples with endometriosis	Moderate
Hong YS et al., 2023 [21]	USA	129	NA	NA	high-risk (16, 18, 26, 31, 33, 35,39, 45, 51, 52, 53, 56, 58, 59, 66, 68, 73, and 82) and low-risk (6, 11, 40, 42, 54, 55, 61, 62, 64, 67, 69, 70, 71, 72, 81, 82, 83, 84, 89, and IS39) types34	Women between 20 and 54 years of age BMI (kg/m2): NA	Reproductive health questionnaire (Sel-report)	A cervicovaginal sample was self-collected	NA	HPV 36.9% high-risk HPV 19.8%	High
Moslehi Z et al., 2023 [27]	Iran	81	NA	Deep endometriosis (DE) Ovarian endometrioma (OMA)	HPV 6 and HPV 11, (the most common types of HPV in endometriosis Patients).	Age (HPV infected): 35*:*65 ± 6*:*91 BMI (kg/m2): 25*:*2 ± 3*:*59	laparoscopic surgery The stage of disease was scored based on the rASRM classification	Before the onset of laparoscopic surgery, a sample of the exocervix was collected from the cervical mucus to examine the presence of HPV. HPV Direct Flow CHIP: PCR assay, followed by reverse dot-blot automatic hybridization based on the DNA Flow Technology	NA	HPV 6: 35% HPV 11: 30% The prevalence of HPV was 24.69%, and low-risk genotypes were dominant	Moderate
Oppelt P et al., 2010 [22]	Germany	56	NA	A total of 66 endometriosis lesions were analyzed from 56 patients including peritoneal (n = 49), ovarian (n = 16), and endometrium (n = 1), and in several patients more than one lesion was analyzed	HPV low-risk types (types 6, 11, 42, 43, and 44) or HPV high-risk types (types 16, 18, 31, 33, 35, 39, 45, 51, 52, 56, 58, 59, and 68)	NA BMI (kg/m2): NA	tissue samples American Fertility Society	HPV DNA: PCR	NA	High-risk: 11.3% medium-risk HPV: 13.2%	Moderate
Hsu LC et al., 2020 [29]	Taiwan	NA	11,198	NA	NA	Women between 15 and 45 years of age. BMI (kg/m2): NA	ICD-9-CM	(ICD-9-CM code 079.4, 078.1, 795.05, 795.09, 795.15, 795.19, 796.75, or 796.79) between 2000 and 2012 as the study cohort and patients without a history of HPV as the control cohort.	4%	The HPV cohort had a higher risk of infertility. The adjusted HR (aHR) was found to be 1.39 (95% CI = 1.19–1.63) after adjusting for demographic characteristics and relevant co-morbidities.	High
Hsu LC et al., 2022 [30]	Taiwan	NA	1136	NA	ICD-9 codes 079.4, 795.05, 795.09, 795.15, 795.19	Women between 15 and 45 years of age. BMI (kg/m2): NA	NA	Patients with a minimum of one inpatient admission or three outpatient visits for HPV were selected	89 (7.83%)	NA	High
Zullo F et al., 2023 [28]	Italy	NA	326 underwent their first IVF cycle and were included in the analysis on IVF results.	NA	HC2 test is a nucleic acid hybridization assay with signal amplification for qualitative detection of 14 HPV types in cervical cells (HPV16, -18, −31, −33, −35, −18, −39, −45, −51, −52, −56, −58, −59, and −68).	HPV+: 36.4 ± 3.7 HPV-: 36.5 ± 3.6 BMI, HPV+: 21.8 ± 3.3 BMI, HPV-: 22.5 ± 3.8	NA	For those who resulted positive for HPV in the cervical swab, HPV genotyping and cervical cytology (PAP test) were performed. Moreover, in case of cervical positivity, the presence of HPV-DNA was also tested in endometrial cells (collected during a mock embryo-transfer before starting IVF procedure) and in granulosa cells (collected from the follicular fluid at the time of oocyte retrieval).	endometriosis was significantly more frequent in HPV-positive than in negative women (31.6% vs. 10.1%; p < 0.01)	8.9% of women candidate to IVF were HPV-positive, HPV16 being the most prevalent genotype.	Moderate
Azizvakili F [23]	Iran	40	The tissues required for testing were obtained through laparoscopy or laparotomy. These samples included cases in which endometriosis lesions were confirmed by a pathologist.	Herpes virus detection was performed using a dedicated diagnostic kit (DNA Technology, Moscow, Russia), HSV1, 2. PCR was performed according to the protocol included in the kit.	Detection of papillomavirus using primers MY09, MY11 related to the L1 gene of the virus using PCR technique	Age: 32.9 ± 5.74 BMI (kg/m2): 22.8 ± 2.75	NA	PCR	Herpesvirus infection in 5 samples (12.5%) of endometriotic tissue and 2 samples (5%) of the control group	Papillomavirus infection, DNA of this virus in 1 sample (2.5%) of tissues from the patient group and 6 samples (15%) of healthy tissue	Moderate
Wei S et al., 2022 [31]	China	NA	224 highrisk HPV (hrHPV) (n = 130) and low-risk HPV (lrHPV) groups (n = 94),	NA	detected 27 genotypes: high-risk genotypes 16, 18, 26, 31, 33, 35, 39, 45, 51, 52, 53, 56, 58, 59, 66, 68, and 82 as well as low-risk genotypes 6, 11, 40, 42, 43, 44, 55, 61, 81, and 83.	HPV-positive (**hrHPV**)women (mean age): 31.7 ± 4.3 HPV-positive (**lrHPV**)women (mean age): 33.7 ± 5.1 HPV-negative women (mean age): 31.9 ± 4.3 BMI (**hrHPV**): 22.8 ± 3.0 BMI (**hrHPV**): 22.6 ± 3.1 BMI- HPV Negative: 23.1 ± 3.5	NA	Regular blood tests were conducted to exclude HIV, HBV, HCV, and TP. All patients underwent a fresh-cycle embryo transfer or a frozen-embryo cycle embryo transfer after the COH cycle. The average transferred embryo number was 1.3 per cycle.	4 (1.8)	The prevalence of HPV infection was 9.2% (747/8117). Of the 747 cases infected with HPV, 529 showed hrHPV infection (70.82%; primarily genotypes 16, 52, 53, 58, and 59); 175 exhibited lrHPV infection (23.43%; primarily genotypes 6, 43, 44, 55, 61, and 81); and 43 cases were co-infected with hrHPV and lrHPV (5.76%).	Moderate
Vestergaard AL et al., 2010 [24]	Denmark	27	NA	ovaries or the peritoneum	DNA was purified from biopsies and subjected to highly sensitive PCR tests detecting human papillomavirus (HPV) types, the herpes family viruses HSV-1 and -2, CMV, and EBV, and the polyomaviruses SV40, JCV, BKV, KIV, WUV, and MCV.	The mean age (±SD) of the participants were 31.72 (±6.29) years for the Endometriosis group and 36.45 (±6.17) years for the Control group. BMI: NA	American fertility score	Cervical smears were tested for the presence of HPV. HPV DNA: PCR	NA	The prevalence of pathogenic DNA viruses in the human endometrium were generally low (0–10%).	Moderate
Rocha RM et al., 2019 [13]	Brazil	29	NA	NA	HPV16 individually, HPV18 individually, and a pool of 12 other hrHPV types (11 definite high-risk, cancer-associated HPV types [HPV31, 33, 35, 39, 45, 51, 52, 56, 58, 59, 68] plus one possibly hrHPV type [HPV66])	Age range of 28 to 47 years and a mean age and SD of 37.25 ± 5.49 years. BMI: NA	American fertility score	The HPV-positive samples were typed by PCR-restriction fragment length polymorphism (PCR-RFLP) analysis, in which amplified DNA was cleaved with restriction enzymes to generate DNA fragments of different molecular sizes.	The endometriosis group was associated with hrHPV positivity in the LGT and UGT sites (P = 0.0002 and P = 0.03, respectively).	Sixty per cent of patients were positive for DNA-HPV in some of the genital tract sites sampled Infertile patients were associated with high-risk HPV (hrHPV) positivity in the UGT sites (P = 0.027).	High
Okyay E et al., 2023 [25]	Turkey.	410	HPV-positive (n = 202) and HPV-negative (n = 208)	NA	14 HPV genotypes (hrHPV) including HPV16, 18, 31, 33, 35, 39, 45, 51, 52, 56, 58, 59, 66, and 68 were investigated. While expressing the HPV results, the term “Other HPV” was used except for HPV 16 and 18, which was referred to as HPV 16/18.	Age: 37.1 *±* 6.3 BMI: NA	Endometriosis symptoms	HPV-DNA from vaginal swab samples	The ovarian endometrioma rate was slightly higher in group HPV 16/18 positive population (16.9%) than in “Other HPV” types positive (11.4%), and HPV-negative groups (7.2%; p = 0.08).	HPV”-positive group (12.8%) was statistically higher than in the HPV-negative group (4.8%; p = 0.007). The infertility rate was significantly higher in the HPV 16/18 positive group (high-risk HPV) 35.8% than in the HPV-negative (7.6%), and “Other HPV” positive group (8%; p < 0.001). Endometriosis-related pain symptoms were significantly higher in high-risk HPV (49%) than in the HPV-negative (37%), and “Other HPV” positive group (46.3%; p = 0.046).	Moderate

PCR: Polymerase Chain Reaction; DE: Deep endometriosis; OMA: Ovarian endometrioma; rASRM: revised American Society for Reproductive Medicine; ICD-9-CM: International Classification of Disease, Ninth Revision, Clinical Modification.

The common high-risk HPV types reported in studies include 16, 18, 31, 33, 35, 39, 45, 51, 52, 56, 58, 59, 66, 68, 73, and 82. The common low-risk HPV types in studies include 6, 11, 30, 34, 40, 42, 43, 44, 54, 55, 61, 62, 70, 72, 74, 81, 83, 84, and 91. Table 3 shows the summary of HPV genotypes by risk category.

**Table 3:** Summary of HPV genotypes by risk category.

First Author	Sample Type	Test/Kit	HPV genotype	The International Agency for Research on Cancer (IARC)
Heidarpour	Tissue-based	polymerase chain reaction/Roche Diagnostics GmbH	High risk	16, 18, 31, 33, 35, 39, 45,52, 56, 58, and59
			Low risk	Types Not reported
Hong	vaginal swab	Roche Molecular Diagnostics, Indianapolis, IN	High risk	16, 18, 26, 31, 33, 35,39, 45, 51, 52, 53, 56, 58, 59, 66, 68, 73, and 82
			Low risk	6, 11, 40, 42, 54, 55, 61, 62, 64, 67, 69, 70, 71, 72, 81, 82, 83, 84, 89
Moslehi	Cervical spatula And tissue biopsy	polymerase chain reaction	High risk	16, 18, 35, 40, 51, 52, 53, 68
			Low risk	6, 11
Oppelt	Tissue-based	polymerase chain reaction/Digene, Hilden, Germany	High risk	16, 18, 31, 33, 35, 39, 45, 51, 52, 56, 58, 59, and 68
			Low risk	6,11,42,43, and44
Hsu [1]	Not reported	Not reported	High risk	Not reported
			Low risk	Not reported
Hsu [1]	Not reported	Not reported	High risk	Not reported
			Low risk	Not reported
Zullo	PAP test, endometrial cells sampling	polymerase chain reaction/Qiagen, Hilden, Germany	High risk	16, 18, 31, 33, 35, 18, 39, 45, 51, 52, 56, 58, 59, and 68
			Low risk	Not reported
Azizvakili	Tissue-based	polymerase chain reaction/Roche, GmbH	High risk	Not reported
			Low risk	Not reported
Wei	Cervical discharges were swabbed	BioRad 100 Amplification and Luminex® 200™ System (Thermo, Waltham, MA, USA)	High risk	16, 18, 26, 31, 33, 35, 39, 45, 51, 52, 53, 56, 58, 59, 66, 68, and 82
			Low risk	6, 11, 40, 42, 43, 44, 55, 61, 81, and 83
Vestergaard	endometrial or lesion tissue samples	polymerase chain reaction/Tissue Kit (Qiagen, Hilden, Germany)	High risk	35,68, 70
			Low risk	90
Rocha	Vaginal and endocervical samples peritoneal fluid and biopsy tissue	polymerase chain reaction/Axygen, CA, USA	High risk	16, 18, 31, 33, 35, 39, 45, 51, 52, 53, 56, 58, 59, 66, 68, 73 and 82
			Low risk	6, 11, 30, 34, 40, 42, 43, 44, 54, 55, 61, 62, 64, 67, 69, 70, 72, 74, 81, 83, 84 and 91
Okyay	vaginal swab samples	Roche Molecular Systems, Pleasanton, CA, USA	High risk	16, 18, 31, 33, 35, 39, 45,51, 52, 56, 58, 59, 66, and 68
			Low risk	Not reported

The included studies used various methods of diagnosis for EM such as laparoscopy, ICD codes, self-reports and using international guidelines. Moreover, HPV detection among included studies was based on various methods like PCR, cytology, and ICD codes.

Different diagnostic approaches for EM such as laparoscopic surgery and verified histologically, questionnaire, ultrasound findings, International Classification of Disease codes were applied. Also, HPV DNA genotypes and polymerase chain reaction (PCR) technique and International Classification of Disease codes were applied for the diagnosis of HPV.

### 3.1 Quality assessment

Of all included studies, eight had moderate quality, and 4 were high-quality studies. Supplementary Tables 1–3 shows the details of quality assessment.

Prevalence findings are now presented in a separate subsection, focusing solely on pooled estimates.

### 3.2 Prevalence of HPV among women with endometriosis

**Fig. 2:**
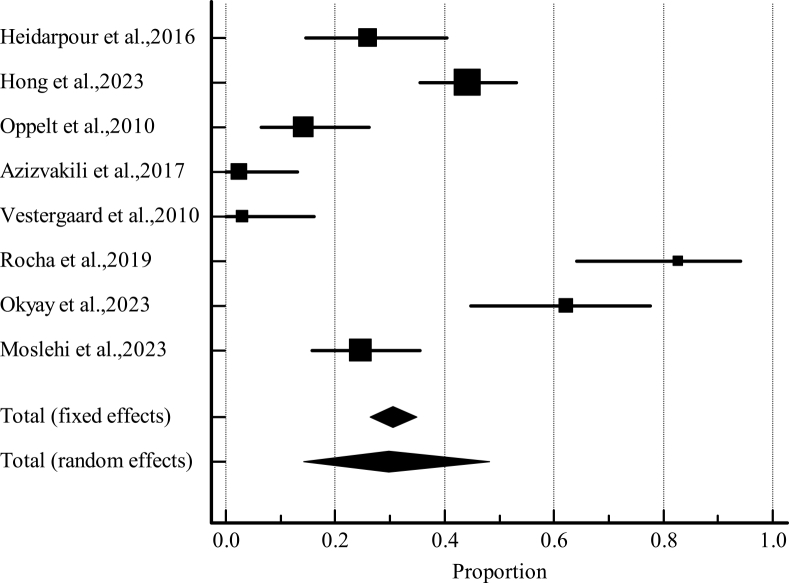
Pooled prevalence estimates of human papilloma virus in women with endometriosis.

The pooled prevalence estimates of HPV regardless of the type among women with EM was 29.73% [95% confidence interval (CI): 14.218 to 48.149; I^2^:93.93%, n = 8 studies). Fig. 2. Publication biases were not significant (Table 4).

**Table 4:** Publication bias of included studies.

Variable	Egger's test	Begg's test
Prevalence of HPV among women with endometriosis	P = 0.7752	P = 0.8046
women with HPV at risk of EM	P = 0.3489	P = 0.1742
Are women with EM at risk of HPV?	P = 0.9292	P = 0.8806

After removing outlier proportions (Rocha et al. and Okay et al.), the pooled prevalence estimates of HPV regardless of the type among women with EM was 18.030% [95% CI: 6.986 to 32.780; I^2^:91.13%, n = 6 studies).

Measures of association derived from the meta-analytic models are reported in a subsequent subsection.

• **Are women with HPV at risk of EM?**

**Fig. 3:**
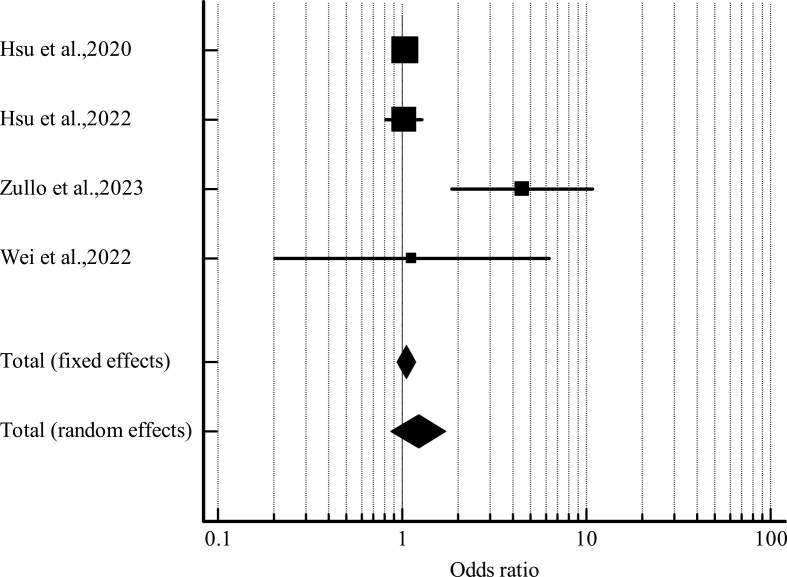
Forest plot of the odds ratio of endometriosis in women with positive human papilloma virus compared to the women with negative human papilloma virus.

Using a random-effects model, the estimated odds ratio of EM in women with positive HPV was 1.226 (95% CI: 0.87–1.724; p = 0.243; I^2^:71.06%, n = 4). Fig. 3. Based on the Egger's test and Begg's test, there was no publication bias among the studies analyzed. (Table 4). After removing study with outlier value (Zullo et al.), the pooled OR also remained non-significant (1.037: 95% CI: 0.921 to 1.168; p = 0.546, I^2^ = 0%, n = 3).

• **Are women with EM at risk of HPV?**

**Fig. 4:**
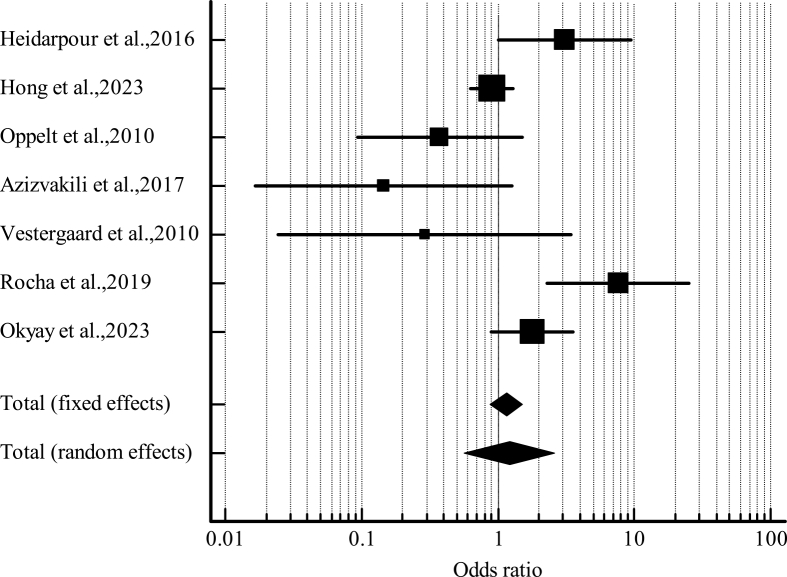
Forest plot of the odds ratio of positive human papilloma virus in women with endometriosis.

The estimated pooled odds ratio of HPV regardless of the type among women with EM was 1.218 (95% CI: 0.569 to 2.608; I^2^ = 73.73%; p = 0.507, n = 7). Fig. 4. In assessing publication bias, there was no significant publication bias. (Table 4).

Due to the limited number of studies (n < 10) included in our meta-analysis, the power of Egger's and Begg's tests to detect publication bias is insufficient. So, these results must be interpreted with caution.

### 3.3 Stratified meta-analysis of HPV-endometriosis association

Table 5 shows the sub-group analyses. In the subgroup examining HPV detection within endometriotic tissue samples from women diagnosed with endometriosis, the pooled prevalence of HPV was 35.7% (95% CI: 13.6%–61.6%). For cervical or vaginal samples collected from women with endometriosis, the pooled prevalence of HPV detection was higher at 52.6% (95% CI: 30.9%–73.8%). Both prevalence estimates were characterized by substantial statistical heterogeneity, as indicated by the wide confidence intervals.

**Table 5:** Pooled analysis in subgroups.

Subgroup	N	Effect size (CI95%)	I^2^
HPV detected in women with EM diagnosed by pathological confirmation (laparoscopy/laparotomy)	7	27.718% (10.569 to 49.239)	94.05%
HPV detected in cervical/vaginal samples of women with EM regardless of diagnosis method	4	52.608% (30.941 to 73.772)	92.04%
HPV detected in tissue samples of women with EM regardless of diagnosis method	4	35.704% (13.641 to 61.606)	93.36%
EM diagnosed via ICD codes in HPV-positive cohorts	2	OR: 1.037 (0.921 to 1.168)	0.0%
EM among n vitro fertilization/intracytoplasmic sperminjection-embryo transfer (IVF/ICSI-ET) candidates screened for HPV	2	OR:2.736 (0.733 to 10.210)	50.82%

In the registry-based cohort analysis focusing on ICD-coded endometriosis diagnoses, individuals identified as HPV-positive showed an odds ratio of 1.037 (95% CI: 0.921–1.168) for the EM diagnosis.

Within the subgroup of in vitro fertilization/intracytoplasmic sperminjection-embryo transfer (IVF/ICSI-ET) candidates, women testing positive for HPV exhibited an odds ratio of 2.736 (95% CI: 0.733–10.210) for EM diagnosis when compared to HPV-negative women. However, the observed association did not reach statistical significance.

## 4 Discussion

The current meta-analysis and systematic review aimed to investigate the potential connection between HPV infection and endometriosis. The pooled prevalence estimates of HPV, regardless of its type, among women with EM were almost 29% (95% CI: 14.21 to 48.14). Given the wide confidence interval, this estimate should be interpreted with caution, as it indicates significant variability in the reported findings across the studies.

The inflammatory milieu plays a crucial role in the development and progression of EM [32]. There is also a close link between the vaginal microenvironment and HPV infection [33]. A recent review proposed that infection is a potential cofactor that contributed to the genetic-epigenetic pathophysiology of EM [34]. HPV can also cause microbial dysbiosis and induce apoptosis in trophoblastic cells, which can disturb the fertility of women [35]. Although some evidence shows EM and infection are likely to coexist, it is unclear whether EM may predispose to HPV and vice versa.

Although our findings showed a higher prevalence of HPV among women with EM, the increased chance of positive HPV among women with EM and vice versa was not observed. A previous study demonstrated that 11% of endometriosis lesions have high and medium-risk HPV infections [34]. This variability may have been caused by several factors, including changes in the study design, sample size, demographic characteristics, regional variations, and laboratory techniques used for diagnosing endometriosis and detecting HPV. Furthermore, because some high-risk HPV subtypes may exhibit distinct behaviors in relation to the pathophysiology of endometriosis, variations in the HPV genotypes examined across studies may have affected the association reported.

The important point is that the included studies applied heterogeneous techniques for the diagnosis of EM and the detection of HPV. Diagnostic approaches for EM ranged from surgical and histopathological confirmation to clinical criteria or even self-report data, while HPV detection methods included PCR-based assays, ICD, and other molecular approaches. This methodological diversity with differing sensitivities and specificities may have led to potential misclassification, particularly in studies relying on non-surgical EM diagnosis or less sensitive HPV assays. Although this heterogeneity could influence the magnitude of the observed associations, it is unlikely to fully account for the overall pattern of results. Nonetheless, future studies using standardized diagnostic criteria and high-sensitivity HPV detection methods are warranted to provide more robust evidence for future guidelines.

Since, a notable degree of heterogeneity was observed across the included studies, we did sup-group analyses. A selected population often facing fertility challenges and undergoing systematic screening with positive HPV have not significantly higher rate of endometriosis. The HPV detection was present in a notable proportion of women with surgically/pathologically confirmed endometriosis. The observed prevalence rates should be viewed as estimates within a heterogeneous landscape rather than absolute values. Future research would significantly benefit from the adoption of standardized outcome measures and more homogeneous study designs to better isolate the true effect size.

Although the NOS scores presented that studies' scores ranged between moderate and high quality, certain limitations were common, including potential selection bias, limited control for confounding factors, and variability in exposure and outcome assessment. Studies may have overestimated or underestimated the true association due to residual confounding or misclassification. Therefore, our pooled estimates should be interpreted with caution, particularly where methodological limitations were present.

There are similarities in pathogenetic mechanisms underlying the bidirectional association between EM and HPV. Anomalies in the inflammatory response in EM cases can impact apoptosis, angiogenesis, extracellular matrix remodeling, and hormonal production [36]. Inflammation, which is caused by immune dysregulation, endocrine system dysfunction, reactive oxygen species (ROS), alterations in epigenetic regulation, carcinogenic pathways, and external environmental factors, including lifestyle, and Dioxins and Polychlorinated Biphenyls (PCBs), are known as mechanisms contributing to the pathogenesis of EM [[37], [38], [39], [40], [41], [42], [43]]. Pathogenesis of HPV Infection also includes chronic inflammation, oxidative stress, cellular processes, and lifestyle [[44], [45], [46]]. Our review revealed a high prevalence of HPV among women with EM within the studied population. When interpreted through the lens of the aforementioned pathophysiological cascade, this high prevalence carries clinical significance. The high prevalence of HPV facilitates a state of chronic microbial dysbiosis, which drives the subsequent immune dysregulation and ROS production observed in these clinical settings.

To better clarify any possible causative link, well-designed, large-scale prospective studies using established diagnostic criteria for both HPV identification and EM categorization are required. Important complicating factors such as sexual behavior, co-infections, parity, hormonal state, and genetic susceptibility should also be taken into consideration in future research. Enhancing knowledge of these connections might have significant clinical ramifications, such as possible prophylactic measures or focused treatment plans for women who are more susceptible to endometriosis.

One of the main limitations of this study was that most of the available evidence was not population-based. In addition, there was considerable heterogeneity across the studies: the viral genotypes investigated were not always the same, and the site of EM lesions varied. Furthermore, the diagnostic approaches used to identify each of the two diseases differed. The significant heterogeneity observed may limit the generalizability of our findings. Future research with a larger number of studies across diverse subgroups would be beneficial to increase the precision of effect estimates. Another limitation of this review is the restriction to English-language publications. This may introduce language bias, as relevant studies have been published in journals featuring other languages. Moreover, limiting the search to PubMed, Scopus, and Web of Science may have introduced a risk of selection bias and may have missed relevant studies indexed in other databases. Future studies suggested to update this review and expand search in other databases like Embase, Cochrane CENTRAL, and CINAHL. The limited search of grey literature and trial registries may have resulted in the exclusion of unpublished or ongoing studies, potentially increasing the risk of publication bias. Readers should have caution, as this summary effect may mask significant underlying differences in risk profiles across distinct clinical settings. Future research with larger, standardized datasets will be essential to delineate these specific associations.

Despite these limitations, this study has some strengths. The inclusion of diverse viral genotypes and lesion sites, although heterogeneous, increases the generalizability of the findings to different populations and clinical contexts. Moreover, the use of various diagnostic methods across studies may strengthen the robustness of the overall conclusions, as it reflects real-world variability in clinical practice rather than a single standardized approach.

## 5 Conclusion

Although this study indicates that a higher prevalence of HPV in women with EM, the pooled odds ratios showed that HPV and EM do not significantly influence the risk of developing each other. Due to the substantial heterogeneity and methodological diversity across the studies, the findings should be interpreted with caution. Consequently, the pooled prevalence estimate should not be cited in isolation, as it may not accurately reflect a single, universal figure, but rather an aggregate of diverse, context-specific data.

Additional research is necessary to investigate the relationship between HPV and EM.

## Ethics approval and consent to participate

Not applicable.

## Availability of data and materials

Not applicable.

## Funding

This study funded by the 10.13039/501100007427Research Institute for Endocrine Sciences, Shahid Beheshti University of Medical Sciences, Tehran, Iran (Grant number: 43017979).

## CRediT authorship contribution statement

**Marzieh Saei Ghare Naz:** Conceptualization, Data curation, Formal analysis, Investigation, Methodology, Software, Validation, Visualization, Writing – original draft, Writing – review & editing. **Mahbanoo Farhadi-Azar:** Methodology, Writing – original draft, Writing – review & editing. **Zahra Marzban Rad:** Writing – original draft, Writing – review & editing. **Shahla Noori Ardebili:** Writing – original draft, Writing – review & editing. **Niloofar Harati:** Writing – original draft, Writing – review & editing. **Niloofar Ghassemi Firouzabadi:** Writing – original draft, Writing – review & editing. **Fatemeh Jayervand:** Writing – original draft, Writing – review & editing. **Fahimeh Ramezani Tehrani:** Conceptualization, Investigation, Methodology, Supervision, Validation, Visualization, Writing – original draft, Writing – review & editing.

## Declaration of competing interest

The authors declare that the research was conducted in the absence of any commercial or financial relationships that could be construed as a potential conflict of interest.

## Abbreviation:

EM = Endometriosis; PID = pelvic inflammatory disease; HPV = human papillomavirus; OR = odds ratio; CI = confidence interval; STI = sexually transmitted infection; ROS = reactive oxygen species; PCBs = Polychlorinated Biphenyls, IVF/ICSI-ET: in vitro fertilization/intracytoplasmic sperminjection-embryo transfer, PCR: polymerase chain reaction.
