# Supplementary material for: Coexistence of endometriosis and human papilloma virus: A systematic review and meta-analysis
Source: New Microbes New Infect. 2026 Jun 19;72:101802. doi: 10.1016/j.nmni.2026.101802 (PMC13320357; doi:10.1016/j.nmni.2026.101802)
Supplement: Multimedia component 1 [file mmc1.docx]

**Supplementary Table 1: Quality assessment of studies cross sectional studies**

| **Author, yer** | **Selection** | | | | **Comparability** | | **Outcome** | | **Overall**  **appraisal** |
| --- | --- | --- | --- | --- | --- | --- | --- | --- | --- |
|  | Representativeness of the sample | Sample size | Non-respondents | Ascertainment of the exposure | Control for the most important factor | control for any additional factor | Assessment of the outcome | Statistical test |  |
| Heidarpour M et al., 2017 | - | - | - | ⁕⁕ | - | - | ⁕⁕ | ⁕ | 5 |
| Hong YS et al., 2023 | ⁕ | ⁕ | - | ⁕ | ⁕ | ⁕ | ⁕ | ⁕ | 7 |
| Moslehi Z et al., 2023 | - | - | - | ⁕⁕ | - | - | ⁕⁕ | - | 4 |
| Wei S et al., 2022 | - | ⁕ | - | ⁕ | ⁕ | ⁕ | - | ⁕ | 5 |
| Vestergaard AL et al., 2010 | - | - | ⁕ | ⁕ | - | - | ⁕ | ⁕ | 4 |
| Okyay E et al., 2023 | ⁕ | ⁕ | - | ⁕ | - | - | ⁕ | ⁕ | 5 |

| Cross sectional studies  Domains | Criteria |
| --- | --- |
| Sample selection (maximum 4 stars) | 1) Representativeness of the sample  a) Truly representative of the average in the target country or region* (all subjects or random sampling)  b) Somewhat representative of the average in the target country or region* (non-random sampling)  c) Selected group of users  d) No description of the sampling strategy |
|  | 2) Sample size  a) pre-determined and calculated*  b) Not pre-determined or calculated. |
|  | 3) Non-respondents  a) Comparability between respondents and non-respondents’ characteristics is established, and the response rate is satisfactory*  (100% response rate)  b) The response rate is unsatisfactory, or the comparability between respondents and non-respondents is unsatisfactory (100%  response rate)  c) No description of the response rate or the characteristics of the responders and the non-responders |
|  | 4) Ascertainment of the exposure (risk factor)  a) Validated measurement tool**  b) Non-validated measurement tool, but the tool is available or described*  c) No description of the measurement tool |
| Comparability(Maximum 2 stars) | 1) The subjects in different outcome groups are comparable, based on the study design or analysis. Confounding factors are  controlled.  a) The study controls for the age (select one)*  b) The study control for other demographic factors* |
| Outcome (Maximum 3 stars) | 1) Assessment of the outcome  a) Independent blind assessment**  b) Record linkage**  c) Self report*  d) No description |
|  | 2) Statistical test:  a) The statistical test used to analyze the data is clearly described and appropriate, and the measurement of the association is  presented, including confidence intervals and the probability level (p value)*  b) The statistical test is not appropriate, not described or incomplete |

**Supplementary Table 2: Quality assessment of studies cohort studies**

|  | **Selection** | | | | **Comparability** | | **Outcome** | | | **Overall**  **appraisal** |
| --- | --- | --- | --- | --- | --- | --- | --- | --- | --- | --- |
|  | Representativeness of the exposed cohort | Selection of the non-exposed cohort | Ascertainment of exposure | Demonstration that outcome of interest | Control for the most important factor | control for any additional factor | Assessment of outcome | Was follow-up long enough for outcomes to occur | Adequacy of follow up of cohorts |  |
| Hsu LC et al., 2020 | ⁕ | ⁕ | ⁕ | ⁕ | ⁕ | ⁕ | - | ⁕ | - | 7 |
| Hsu LC et al., 2022 | ⁕ | ⁕ | ⁕ | ⁕ | ⁕ | ⁕ | - | ⁕ | - | 7 |
| Zullo F et al., 2023 | - | - | ⁕ | ⁕ | - | - | ⁕ | - | ⁕ | 4 |

| Cohort studies | Criteria |
| --- | --- |
| Sample selection (maximum 5 stars) | 1) Representativeness of the exposed cohort  a) truly representative of the average population in the target country or region*  b) somewhat representative of the average population in the target country*  c) selected group of users  d) no description of the derivation of the cohort |
|  | 2) Selection of the non-exposed cohort  a) drawn from the same community as the exposed cohort*  b) drawn from a different source  c) no description of the derivation of the non-exposed cohort |
|  | 3) Ascertainment of exposure  a) secure record*  b) structured interview*  c) written self-report  d) no description |
|  | 4) Demonstration that outcome of interest was not present at start of study  a) yes*  b) no |
| Comparability(Maximum 2 stars) | 1) Comparability of cohorts on the basis of the design or analysis  a) study controls for age ⁕  b) study controls for other demographic factors*⁕ |
| Outcome (Maximum 3 stars) | 1) Assessment of outcome  a) independent blind assessment*  b) record linkage*  c) self-report  d) no description |
|  | 2) Was follow-up long enough for outcomes to occur  a) ≥6 months *  b) <6 month |
|  | 3) Adequacy of follow up of cohorts  a) complete follow up - all subjects accounted for  b) subjects lost to follow up unlikely to introduce bias - small number lost - 100 % follow up, or description provided of those lost)  c) follow up rate < 100% and no description of those lost  d) no statement |

| **Author, year** | **Selection** | | | | **Comparability** | | **Exposure** | | | **Overall** |
| --- | --- | --- | --- | --- | --- | --- | --- | --- | --- | --- |
|  | Is the case definition adequate? | Representativeness of the cases | Selection of Controls | Definition of Controls | Control for the most important factor | control for any additional factor | Ascertainment of exposure | Same method of ascertainment for cases and controls | Non-Response rate | **appraisal** |
| Oppelt P et al., 2010 | ⁕ | - | - | ⁕ | ⁕ | - | - | - | ⁕ | 4 |
| Azizvakili et al., 2017 | ⁕ | - | - | ⁕ | - | - | ⁕ | ⁕ | - | 4 |
| Rocha RM et al., 2019 | ⁕ | ⁕ | - | ⁕ | ⁕ | ⁕ | ⁕ | ⁕ | - | 7 |

**Supplementary Table 3: Quality assessment of studies case control studies**

| Case-control studies  Domains | Criteria |
| --- | --- |
| Sample selection (maximum 5 stars) | 1) Is the case definition adequate?  a) yes, with independent validation*  b) yes, e.g. record linkage or based on self-reports  c) no description |
|  | 2) Representativeness of the cases  a) consecutive or obviously representative series of cases*  b) potential for selection biases or not stated |
|  | 3) Selection of Controls  a) community controls*  b) hospital controls  c) no description |
|  | 4) Definition of Controls  a) no history of disease (endpoint) *  b) no description of source |
| Comparability(Maximum 2 stars) | 1) Comparability of cases and controls on the basis of the design or analysis  a) study controls for age, and sex*  b) study controls for other demographic factors* |
| Exposure (Maximum 3 stars) | 1) Ascertainment of exposure  a) secure record*  b) structured interview where blind to case/control status*  c) interview not blinded to case/control status  d) written self-report or medical record only  e) no description |
|  | 2) Same method of ascertainment for cases and controls  a) yes*  b) no |
|  | 3) Non-Response rate  a) same rate for both groups*  b) non respondents described  c) rate different and no designation |
